# Reaction Time of Motor Responses in Two-Stimulus Paradigms Involving Deception and Congruity with Varying Levels of Difficulty

**DOI:** 10.1155/2005/804026

**Published:** 2005-08-04

**Authors:** Jennifer M. C. Vendemia, Robert F. Buzan, Stephanie L. Simon-Dack

**Affiliations:** Department of PsychologyUniversity of South CarolinaColumbiaSCUSA

## Abstract

Deception research has focused on identifying peripheral nervous system markers while ignoring cognitive mechanisms underlying those markers. Cognitive theorists argue that the process of deception may involve such constructs as attentional capture, working memory load, or perceived incongruity with memory, while psychophysiologists argue for stimulus salience, arousal, and emotion. Three studies were conducted to assess reaction time (RT) in relation to deception, response congruity, and preparedness to deceive. Similar to a semantic verification task, participants evaluated sentences that were either true or false, and then made truthful or deceptive evaluations of the sentence’s base truth-value. Findings indicate that deceptive responses have a longer RT than truthful responses, and that this relationship remains constant across response type and preparedness to deceive. The authors use these findings in preliminary support of a comprehensive cognitive model of deception.

## 1. Introductory

Deception is defined as the "act of deliberately providing or omitting information with the intention of misleading" [22]. Deception research has focused on identifying peripheral nervous system markers and has ignored cognitive mechanisms underlying those markers. Understanding the cognitive processes involved in deception will help to build a better lie detector, one not subject to the false-negatives and inconclusive findings possible in traditional polygraph techniques. The field of lie detection has languished in part because researchers have not worked from a theoretical framework, instead choosing to indulge in a data-driven approach to the problem. While such data-driven approaches are useful in solidifying the connections between the act of deception and its concomitant physiological responses, they do not provide the information necessary to improve the field of lie detection beyond a certain ceiling of reliability [13,17,29,35]. Not only does theory-driven research provide the opportunity to improve lie-detection techniques, it enhances the ability of researchers from varied traditions to investigate the same problem.

## 2. Background research

Most reaction time (RT) studies of deception were conducted in the 1920s and 1930s and have not been revisited [7,11,16,23,24,30–32]. In 1905 and 1906, Wertheimer and Jung, working separately, both discovered that Galton's Word Association Test could be used to identify emotional complexes relating to guilt [16, 48], and applied this particular method to study crime detection. This research showed that RT was greater when criminals attempted to deceive researchers by avoiding incriminating questions [16].

Marston conducted the first studies pertaining to RT and concluded that deception could cause either increases or decreases in RT [31–33]. Other researchers argued that the decreases Marston reported in RT had been due to confounding non-deceptive responses that

ISSN 0953-4180/04/$17.00 © 2004 – IOS Press and the authors. All rights reserved occurred when they were unaware that their statements were false [11,16]. All participants' RTs increased while they were aware that they were lying. Because of these studies, the "awareness" of prevarication has become central to its definition.

Presently, many researchers in the field of detection of deception use the "Concealed Information Test" (CIT [22]) formerly named the "Guilty Knowledge Task" (GKT [23–28]). The CIT consists of a single item related to the crime positioned among a group of items not related to the crime. The recognition of the crime-related information causes a variety of peripheral nervous system responses that are easily measured by polygraph, such as changes in respiration, increases in galvanic skin response, and changes in blood pressure. Although the CIT is an excellent test when used in the appropriate situation, such as questioning a suspect in a single-issue crime for which there are no witnesses, it is often not applicable to real-world situations [5,22]. Additionally, independent research suggests that the CIT may be predisposed to false negatives (deceptive examinees scored as truthful [25,29,36]). Furthermore, some concern has been noted that the test results may be heavily confounded by episodic memory [1]. In fact, the Psychophysiological Detection of Deception field has replaced the term "guilty knowledge" with the term "concealed information" to reflect the fact that in the CIT, the peripheral nervous system response is not correlated with guilt, but rather with possession of knowledge. For example, witnesses of a crime would have the same knowledge as the person who committed the crime and would have the same type of peripheral nervous system response [22].

Few studies of RT relating to deception have been conducted since 1950. Of these studies, most have used the CIT paradigm [1,14,15,41,43]. Seymour et al. [43], in a series of experiments, had participants commit a mock computer crime, and then respond to probes containing irrelevant words and probes consisting of crime-related words. The crime-related probes were presented 17% of the time. In the first experiment participants were to respond to probe items without being deceptive, while in the last two studies they were instructed to be deceptive. Across studies, participants who possessed concealed information exhibited longer RTs to the crime-related probe items regardless of intent to deceive. No difference was found in participants who did not possess concealed information. This study demonstrates that possession of concealed knowledge increased RT independent of the participants' attempts to be deceptive. Seymour's [43] findings are confounded by the fact that the crime-relevant probes were presented infrequently among more frequently occurring non-target stimuli. Jones et al. [20] evaluated the effects of infrequently occurring stimuli and found that low frequency led to longer RTs.

## 3. Theoretical approaches to deception

Multiple competing theories of deception exist. Cognitive theorists argue that the process of deception may involve such constructs as attentional capture [2], working memory load [8], or perceived incongruity with semantic and episodic memory [6], while psychophysiologists argue for the importance of stimulus salience, arousal, and emotion [17]. Figure 1 depicts our proposed model of deception based on the main constructs that have been historically implicated in the process of lying. This model merges the cognitive and physiological components potentially underlying the act of deception that have been described by the aforementioned researchers. It further suggests the possibility and direction of influences of each construct upon one another, the observation methods most appropriate for measuring these constructs, and provides relationships that are experimentally testable.

Deception-related cognitive activity can be assessed with imaging techniques that examine activity within the central nervous system (CNS), while physiological measures assess the emotional influences of deception. Furthermore, RTs serve as a direct measure of the deceptive or truthful response. Research currently underway in our laboratory uses event-related brain potentials (ERPs) and RTs to examine the cognitive activities described in the model. The current series of studies evaluates the RTs associated with truthful and deceptive responses.

Two main cognitive theories based on the timing of the P3b waveform within ERP studies, the attention theory and the working memory load theory, suggest different patterns of response for the P3b based on the antagonistic effects of attention and working memory load ("workload" [21]). Attention theorists argue that attentional capture of the low frequency CIT items (i.e., stimuli appearing infrequently within the experimental block) increases the amplitude of the P3b [12], while workload theorists argue that the increased working memory demands required for deceptive processing suppresses the P3b. Deception is one of the most complex social behaviors in which humans engage. It is unlikely that any single construct explains the process

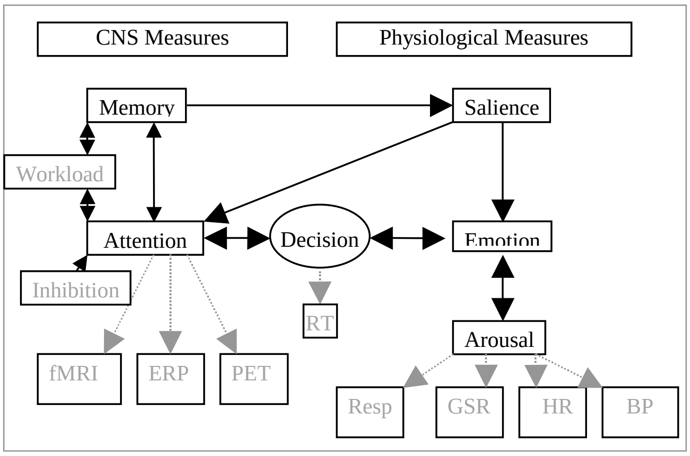

A proposed model of the process of deception. CNS measures such as fMRI, ERP, PET tend to emphasize paradigms that focus on memory and attention, while paradigms involving respiration, GSR, heart rate, and blood pressure tend to emphasize paradigms that manipulate emotion, arousal, and stimulus salience. (fMRI = functional magnetic resonance imaging, ERP = event-related potential, PET = positron emission tomography, RT = reaction time, Resp = respiration, GSR = galvanic skin response, HR = heart rate, BP = blood pressure)

Fig. 1. Proposed relationship of the underlying theoretical constructs involved in the process of deception.

of deception. As our comprehensive model (Fig. 1) shows, motivation, emotion, attention, arousal, and memory all interact during any act of deception; no single study can explore all of these components at one time.

Deception is seen as a process requiring greater cognitive effort than truth telling [47]. Responding truthfully requires only that one evaluate the stimulus questions in comparison with known information [6]. Deception, on the other hand, requires the same comparison but with a deliberate modification of the response. Although attention, motivation, arousal, and memory contribute to the decision process, it is the increased workload demands brought about by the additional task of modifying a known truthful answer into a deceptive one that appears to correlate with RT [45].

An alternative to the CIT is the “Comparison Question Technique” formerly known as the “Control Question Technique” (CQT [3,10,38]). This technique relies on manipulating the salience of specific test questions during the pre-test interview by placing examinees unwittingly in a Hobson’s Choice dilemma. A Hobson’s Choice dilemma is a situation in which the participant feels that deception is the only option [44]. To avoid the influence of the Hobson’s Choice dilemma, the current study used a specific form of the comparison question test, called the “directed lie comparison test” (DLC). In the DLC, participants are instructed to lie to specific questions throughout the exam. The DLC is divided into two main categories dependent on question content: (1) Trivial DLC, in which respondents lie to non-relevant trivial items and (2) personally significant DLC, in which respondents lie to items of some relevance to themselves [22]. The DLC is more standardized than other forms of the CQT; it requires less psychological manipulation; it is less intrusive to participants; and it has been described as easier to explain in court [37]. Although there is some debate on the topic [4,39], the validity of the test has been established in laboratory [19] and field [18] studies using traditional polygraph measures. The paradigm that we used throughout this series of experiments was a form of the trivial DLC.

## 4. Current research

A shortcoming of much of the extant literature is that research has measured the possession of concealed knowledge, rather than deception per se. Lacking a theoretical model of deception, researchers have conducted data-driven research, which promotes the study of concealed information instead of deception. The current research overcomes this by evaluating the psychophysiological consequences of deception in which there is no concealed information. Additionally, all stimuli were presented equiprobably. This allowed the effects of attention and workload to be parametrically equated on a trial-by-trial basis independent of stimulus presentation probability. Furthermore, the equiprobable nature of the relevant and irrelevant stimuli eliminated the potential for any RT findings to be confounded by stimulus frequency.

The goal of this series of studies was to isolate constructs within the postulated theory of deception, and manipulate variables related to those constructs. In these experiments, we manipulated variables related to attention and task workload. We controlled memory by including sentences with semantic memory information or generic factual information, and balanced memory access by having participants respond truthfully and deceptively to each question an equal number of times. The paradigm we used was similar to a semantic verification task [34]. In a semantic verification task, participants verify whether a proposition is true or false. In our framework, we added a deception condition to the semantic verification task.

Within each experiment, we balanced factors known to influence stimulus salience including redundancy, illumination, and modality [9,42]. Additionally, we balanced the number of deceptive and truthful cues. To the extent that it was possible, arousal and emotion constructs were held constant throughout the studies. Stimulus relevance, another factor known to influence salience [42], was systematically increased across the three tasks. The anticipated effect of doing so was to increase the salience of the target stimulus across the studies.

As theoretically driven research, the current work speaks to questions of workload and attention. We anticipate that the theoretical model of deception will expand from its workload base to include issues of memory, salience, emotion, and arousal. Through our work, we expect to develop a comprehensive model of deception that researchers from various traditions will find useful in their divergent explorations of the deception process.

## 5. Methods

Three studies of increasing difficulty were conducted to assess RT in relation to deception, response congruity, and preparedness to deceive. Participants were asked to evaluate sentences (Stimulus 1) that were either true or false, compare those evaluations with a second stimulus (either “true” or “false”), and respond truthfully or deceptively. The goal of these studies was to assess the effects of deceptive and truthful responses on RT while accounting for additional RT variability associated with response congruity and preparedness. In Experiment 1, all the information needed for participants to correctly complete the task was presented within Stimulus 1. In Experiment 2, information regarding deception was available from the first stimulus, but information regarding response congruity was not available until the onset of Stimulus 2. By Experiment 3, the predictability of Stimulus 2 from Stimulus 1 was reduced to zero, increasing the amount of information to be absorbed from Stimulus 2. This resulted in greater salience and workload demands as the predictive value of Stimulus 1 decreased. Table 1 shows the predictability of response type across the three studies.

Table 1
First stimulus cues for response type (deceptive, truthful, congruent, incongruent) across the three studies

| | Predictability of response from first stimulus | |
|---|---|---|
| | Deception | Congruity |
| Experiment 1 | + | + |
| Experiment 2 | + | − |
| Experiment 3 | − | − |

+ indicates presence of cue, − indicates lack of cue.

As derived from the model in Fig. 1, we expected that increased workload demands would lead to increased RTs for deceptive responses than for truthful responses within each experiment. Moreover, incongruent responses were expected to take longer than congruent responses within each experiment. Across studies, we expected that decreased preparedness to lie would increase the salience of the second stimulus, forcing participants to allocate more attentional resources to the second stimulus in order to obtain the information necessary to formulate their response. Therefore, we expected workload demands to increase across studies as participants were required to make more decisions at the onset of Stimulus 2, resulting in longer RTs. We predicted that the increased attention would result in decreased RT, but that the effects due to workload would supersede those due to attention. In our model of deception, attention functions primarily as a mediator variable between workload and decision as measured by RT. However, based on pilot research, we expected its impact to be minimal. The within-experiment results will be presented following the procedure for each experiment, while the across-paradigm manipulation results will be presented following Experiment 3.

Table 2
Example of sentences used in the three experiments

| Base truth value | Stimulus sentence |
|---|---|
| True | The grass is green. |
| | South Carolina is in the united states. |
| | Ducks spend most of their time in the water. |
| | A piano is a musical instrument. |
| | Poodles are dogs. |
| False | Snakes have 13 legs. |
| | People are born wearing clothes. |
| | The slowest runner always wins the race. |
| | Cupcakes are healthier than salad. |
| | President George Washington cleans my kitchen. |

### *5.1. Experiment 1*

#### *5.1.1. Participants*

Participants were 45 undergraduate students (26 women, 19 men) recruited from the University of South Carolina student population. Their ages ranged from 18–43 (M = 21.38, SD = 4.24). All had normal or corrected to normal vision with no known color impairments and were right handed. Participants were also screened for a variety of neurological and medical disorders and were asked to avoid drugs, alcohol, and caffeine for 24-hours preceding the experiment. Participants received course credit for their participation.

#### *5.1.2. Task*

Each participant sat in a comfortable chair approximately 122 cm from a 29 color video computer monitor (NEC Multisync XM29) displaying at 1280 horizontal and 1024 vertical pixels.

The two-stimulus paradigm involved the pairing of a first stimulus, which participants evaluated, and a second stimulus to which they responded. Each first stimulus was drawn from a series of 60 sentences involving declarative knowledge that were designed to be easily verified as true or false (e.g., “I am human”). Several examples of the sentences used are shown in Table 2. These stimuli were derived from a set of 100 short, easy to understand sentences that had been pre-tested with an undergraduate sample at the university. Raters were asked to decide whether each sentence was true or false. Only those items unanimously rated as “true” or “false” during pre-testing were retained for the experiments.

Sentence presentation lasted 2500 ms, followed by a 750 ms fixation point, then a second stimulus of 2500 ms duration. Participants responded to the second stimulus by pressing a key to indicate whether it agreed or disagreed with their answer to the first stimulus. RT was defined as the latency between the onset of Stimulus 2 and the participant’s response. This procedure is similar in nature to that of Rosenfeld et al. [40], who used a modified forced-choice procedure to detect malingering.

Participants were cued by sentence color to respond deceptively on 50% of the trials and truthfully on the other 50%. Additionally, participants were required to make a congruent response (i.e., “agree”) on 50% of the trials and an incongruent (i.e., “disagree”) response on the other 50% of the trials. The stimuli were presented in red or blue, the color of Stimulus 1 always matched that of Stimulus 2, and deceptive and truthful trials were randomly presented. Participants were randomly assigned which color cued deception. Furthermore, the color of Stimulus 1 always predicted Stimulus 2. For example, when presented with a red Stimulus 1, a given participant would always receive a red “True” as Stimulus 2. The relationship between color and Stimulus 2 was counterbalanced across participants.

As shown in Fig. 2, when participants were color cued to be truthful and the second stimulus provided an accurate description of the truth state of the first stimulus they responded by pressing “agree”. We labeled this “congruent truthful” to denote that the respondent truthfully indicated that the second stimulus was congruent with their answer to the first stimulus. When color cued to be truthful and the second stimulus did not provide an accurate assessment of the truth state of the first stimulus, they responded by pressing “disagree” (incongruent truthful). When color cued to be deceptive and the second stimulus provided an inaccurate answer, they responded “agree” (congruent deceptive). Finally, when color cued to be deceptive but the second stimulus accurately described the truth state of Stimulus 1, they responded “disagree” (incongruent deceptive). This resulted in four experimental conditions: congruent truthful, congruent lie, incongruent truthful, and incongruent lie (CT, CL, IT, IL). RT and error data were collected on three blocks of 60 randomized trials each. This resulted in 45 trials of each trial type.

#### *5.1.3. Procedure*

Participants arrived at the lab on the day of the experiment and were familiarized with the research procedure before signing the consent form. They practiced on a pencil and paper measure that included all stimuli used in the study. Following the paper task, participants were seated in front of the monitor, verbally instructed on the use of the response box, and received additional computer-based practice to train them to respond within the allowed response window of 2500

| Condition | Stimulus 1 (2500 ms) | Fixation (750 ms) | Stimulus 2 (2500 ms) | Response |
|---|---|---|---|---|
| Congruent Truthful | *The grass is red* | + | *False* | Agree |
| Incongruent Truthful | *The grass is green* | + | *False* | Disagree |
| Congruent Deceptive | **The grass is red** | + | True | Agree |
| Incongruent Deceptive | **The grass is green** | + | True | Disagree |

Note: In this example, the italicized text cues truthful responding, while the boldfaced text cues deceptive responding. A congruent response is one in which the participant indicates that Stimulus 2 is congruent with (i.e., "agrees" with) his or her answer for Stimulus 1.

Fig. 2. Time course of stimulus administration for the two-stimulus paradigm in Experiment 1.

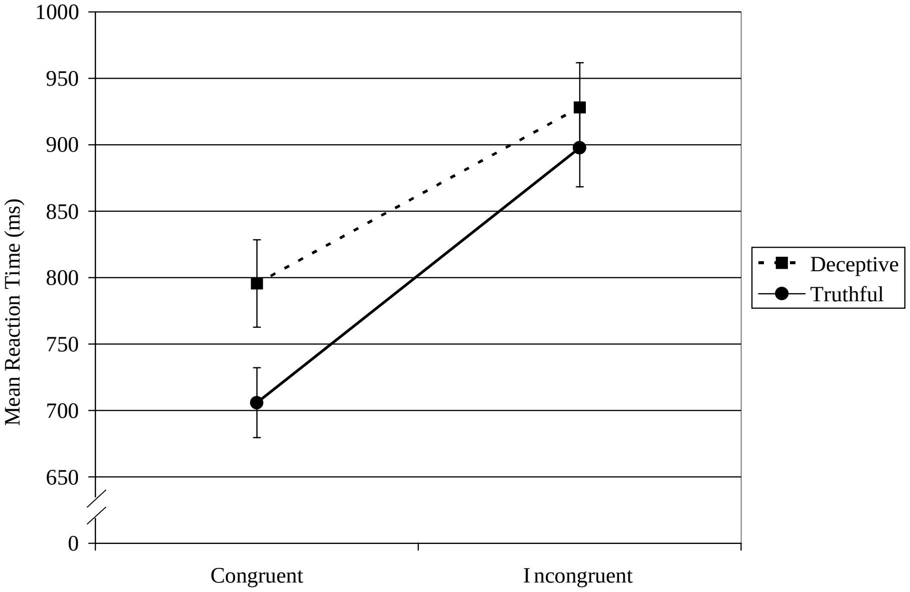

Fig. 3. Mean reaction time in a two-stimulus paradigm in which both response deception and response congruity were predictable in Experiment 1.

ms. The computer-based practice consisted of 12 items from the larger block of questions, constrained so that it contained equal numbers of CL, CT, IL, and IT questions. Participants were required to attain a 67% accuracy level on each of the trial types in order to begin the experiment. Records of the number of practice block repetitions that were required and the time to completion were not kept, but six participants were disqualified from further participation because they could not achieve the 67% correct threshold. During the experiment, participants initiated each trial by key press. They were instructed to rest during the period between trials if they felt tired. During the rest period, the stimulus presentation screen reminded participants of the response box instructions.

### 5.1.4. *Results*

Due to the hypothesized differences in difficulty between conditions, we expected that respondents would commit more errors on deceptive trials than on truthful trials and more errors on incongruent trials than congruent trials. No significant differences were found in the error rates between trial types.

In order to assess the impact of deceptive responding and response congruity, a 2 $\times$ 2 (deception $\times$ response congruity) ANOVA was performed using the RT data. Main effects and an interaction were identified for deception and response congruity. As Fig. 3 shows, participant RTs were significantly longer for deceptive than truthful responses $F(1,44) = 7.72$, $p = 0.008$ and significantly longer for incongruent than congruent re-

| Condition | Stimulus 1 (2500 ms) | Fixation (750 ms) | Stimulus 2 (3000 ms) | Response |
|---|---|---|---|---|
| Congruent Truthful | *The grass is green.* | + | *True* | Agree |
| Incongruent Truthful | *The grass is green* | + | *False* | Disagree |
| Congruent Deceptive | **The grass is green** | + | **False** | Agree |
| Incongruent Deceptive | **The grass is green** | + | **True** | Disagree |

Note: In this example, the italicized text cues truthful responding, while the boldfaced text cues deceptive responding. A congruent response is one in which the participant indicates that Stimulus 2 is congruent with (i.e., "agrees" with) his or her answer for Stimulus 1.

Fig. 4. Time course of stimulus administration for the two-stimulus paradigm in Experiment 2.

sponses F(1,44) = 138.11, $p$ = 0.000. Additionally, an interaction occurred such that incongruent deceptive responses had the longest RTs followed by incongruent truthful responses, congruent deceptive responses, and congruent truthful responses F(1,44) = 4.50, $p$ = 0.04.

### 5.2. *Experiment 2*

In the second study, response congruity was not predictable from cues in Stimulus 1. Participants could utilize Stimulus 1 to prepare to lie or tell the truth, but could not predict whether they would do so by agreeing or disagreeing. We expected longer RTs overall, particularly for trials in which participants responded incongruently.

#### 5.2.1. *Participants*

Participants were 44 undergraduate students (24 women, 20 men) recruited from the University of South Carolina student population. Their ages ranged from 18–43 (M = 21.32, SD = 5.31). The screening and incentive procedures were identical to Experiment 1. Four participants were unable to complete the practice trials.

#### 5.2.2. *Task*

The task was identical to the task in Experiment 1 with one exception. In the second study, the first stimulus exclusively predicted deception. Thus, participants would not be able to determine the specific response until the onset of the second stimulus (see Fig. 4). As in all three experiments, deception cue color was randomly assigned.

#### 5.2.3. *Procedure*

The procedure was identical to Experiment 1.

#### 5.2.4. *Results*

In order to assess the impact of deceptive responding and response congruity, a 2 $\times$ 2 (deception $\times$ response congruity) ANOVA was performed using the RT data. Although only deception was predictable based on Stimulus 1 cues, main effects were identified for both deception and response congruity. However, no interaction between these variables was identified. Figure 5 shows that participant RTs were significantly longer for deceptive than truthful responses F(1,43) = 11.88, $p$ = 0.008 and for incongruent than congruent responses F(1,43) = 69.39, $p$ = 0.000. As in Study 1, no differences were seen in error rates between response conditions.

### 5.3. *Experiment 3*

In the third study, Stimulus 1 sentences were colored black, offering no predictive value for either response congruity or the truth-value of the response. Participants could prepare neither to respond deceptively or truthfully nor to agree or disagree in response to the stimuli. We expected RT to be longer overall, and RT to be particularly affected in those conditions in which participants responded deceptively and incongruently.

#### 5.3.1. *Participants*

Participants were 38 undergraduate students (24 women, 14 men) recruited from the University of South Carolina student population. Their ages ranged from 18–43 (M = 20.27, SD = 3.44). The screening and incentive procedures were identical to Experiment 1. The number of participants who could not successfully complete the practice trials was lost due to a computer error.

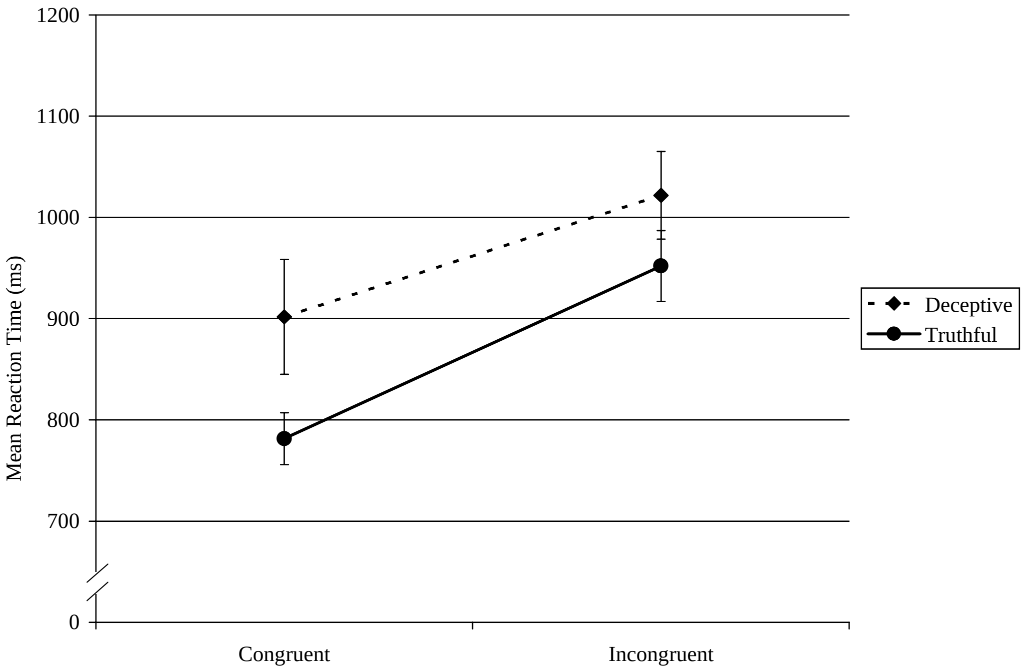

Fig. 5. Mean reaction time in a two-stimulus paradigm in which only response deception was predictable in Experiment 2.

| Condition | Stimulus 1 (2500 ms) | Fixation (750 ms) | Stimulus 2 (3000 ms) | Response |
|---|---|---|---|---|
| Congruent Truthful | The grass is green. | + | *True* | Agree |
| Incongruent Truthful | The grass is green | + | *False* | Disagree |
| Congruent Deceptive | The grass is green | + | **False** | Agree |
| Incongruent Deceptive | The grass is green | + | **True** | Disagree |

Note: In this example, the italicized text cues truthful responding, while the boldfaced text cues deceptive responding. A congruent response is one in which the participant indicates that Stimulus 2 is congruent with (i.e., "agrees" with) his or her answer for Stimulus 1

Fig. 6. Time course of stimulus administration for the two stimulus paradigm in Experiment 3.

*5.3.2. Task*

The task in Experiment 3 differed from the task in the earlier experiments in two ways. In the first study, the first stimulus predicted both deception and response. In the second study, the first stimulus exclusively predicted deception. In the third study, the first stimulus predicted neither deception nor congruity (see Fig. 6). Thus, participants would not be able to predict the nature of the response until the onset of the second stimulus. In addition, the presentation time of the second stimulus was increased to 3000 ms to allow participants enough time to respond. This modification was based on pilot testing, which indicated that participants in this more difficult experiment required more time to generate the correct response.

*5.3.3. Procedure*

The procedure was identical to experiments 1 and 2.

*5.3.4. Results*

A 2 $\times$ 2 (deception $\times$ response congruity) ANOVA was performed using the RT data. Main effects were identified for both independent variables as well as an interaction between them. As Fig. 7 shows, participant

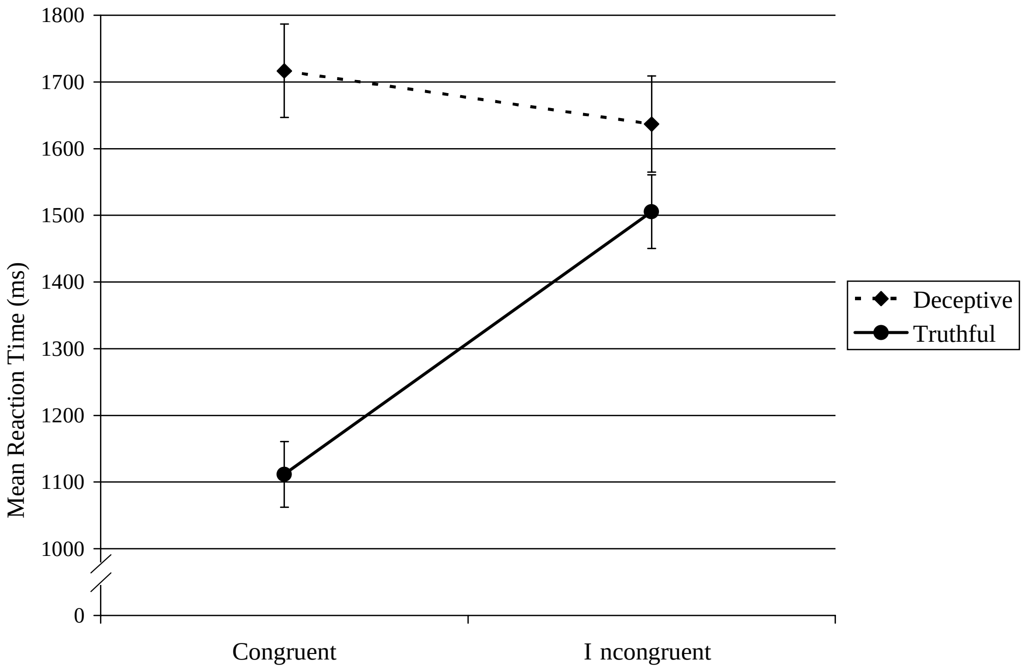

Fig. 7. Mean reaction time in a two-stimulus paradigm in which neither response deception nor response congruity was predictable in Experiment 3.

RTs were significantly longer for deceptive than truthful responses F(1,37) = 75.29, $p = 0.000$ and significantly longer for incongruent than congruent responses F(1,37) = 59.24, $p = 0.000$. Additionally, an unexpected interaction occurred such that congruent deceptive responses had the longest RTs followed in order of average RT by incongruent deceptive responses, incongruent truthful responses, and congruent truthful responses F(1,37) = 60.48, $p = 0.000$. No differences were noted in error rates between response conditions.

## 6. Response predictability across the three experiments

In order to assess the impact of Stimulus 1 cues on participant responses, two multivariate analyses were performed. The first included the error measures for each of the four task conditions across the three studies (CL, CT, IL, IT). The second included RT measures for each of the four conditions. No significant differences were found for error measures; however significant differences were found for the RT measures in each of the conditions. Using the *Wilk's* criterion, a main effect was found for predictability of response type based on the cues from Stimulus 1. Figure 8 shows that as predictability of the second stimulus decreased, RT increased F(8, 42) = 17.84, $p < 0.0001$.

Post-hoc comparisons were performed using the Bonferroni adjustment for multiple comparisons. RT was significantly longer when neither congruity nor deception was predictable M = 914.20 ms (SE = 40.41 ms) than in other conditions.

## 7. Discussion

This series of studies demonstrates that deceptive responding has a longer latency than truthful responding across levels of response preparedness. Regardless of the congruity of participant response or how prepared they were to lie, participants exhibited longer RTs for telling lies than for telling the truth. This study replicates the findings of early researchers [11,16], and supports similar studies using probabilistic paradigms [43].

Research in our laboratory has consistently found reliable differences in RT [45,46]. RTs for deceptive responding are longer than for truthful responding and RTs for incongruent responding are typically longer than for congruent responding. As preparedness to deceive or tell the truth decreases, these differences become more pronounced. The most plausible explanation for these findings involves workload. Congruent responses are less demanding than incongruent responses, and truthful responses are less demanding than deceptive responses. In Experiment 3, however, we

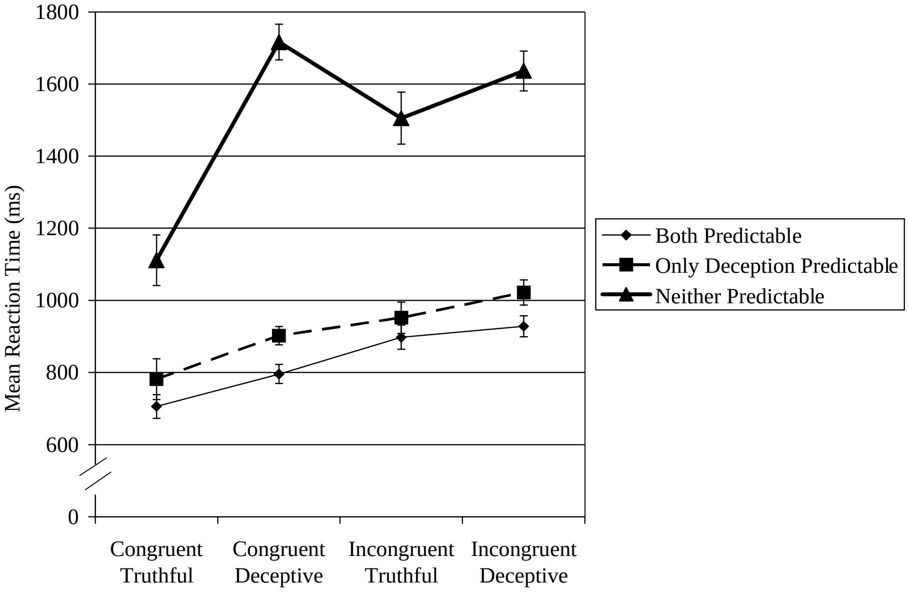

Fig. 8. Summary of mean reaction time scores across two-stimuli tasks with varying degrees of predictability.

found longer RTs for congruent deceptive responding than for incongruent deceptive responding. Although this difference was not significant, it merits consideration as it cannot be explained by the effects of workload alone. The impact of attention, which was greatest in Experiment 3 due to the increased salience of the second stimulus, may very well have mediated the effects of workload on deceptive responding. Thus, these findings could offer support for the relationships between workload, attention, and deceptive responding proposed in our theoretical model of deception, but also suggest further lines of research to expand our understanding of the cognitive foundations of deception. In order to more completely investigate this, additional research has been conducted in our laboratory utilizing personally-relevant autobiographical information, which has more salience to the respondent. The unusual congruity effect was replicated in that study [45].

If differences in task conditions were related exclusively to difficulty in choosing the correct response (as opposed to workload specifically related to deception), one would hypothesize that differences in RT would be the same for both processes involving workload – deception and congruity. In this series of studies, we see greater increases related to deception than congruity. These differences are noted not only in differences in mean RT, but in the pattern of RT differences related to each construct, suggesting that the cognitive processes involved in formulating deceptive responses are different from those involved in formulating a congruent or incongruent response. Further research utilizing more complex relationships between congruity, deception, and workload is planned for our laboratory, with the expectation that such efforts will help to more clearly delineate the cognitive relationships between these constructs.

The current series of studies examined the effect of deception- and response congruity-related workload on RT to explore the underlying cognitive and neural mechanisms of deception. Based on the current findings, we expect to continue evaluating our theoretical model of deception, further exploring the effects of workload on deception as measured by RTs and event-related potentials. However, we also anticipate the expansion of research in our laboratory to evaluate additional constructs within the model, such as attention, memory, salience, emotion, and arousal. Through our work, we expect to develop a comprehensive model of deception that researchers from various traditions will find useful in their divergent explorations of the deception process.

## Acknowledgments

This research was supported by a grant from the Department of Defense Polygraph Institute, # DABT60-00-1-1000, and a Major Research Instrumentation Award, # BCS-9977198. The authors wish to acknowledge Dr. John Richards for his technical advice and support, and William Campbell for his aid in computer program design, testing participants, and data editing.

This research was partially supported by NSF grant 9911132.
